# Mean SOFA Score in Comparison With APACHE II Score in Predicting Mortality in Surgical Patients With Sepsis

**DOI:** 10.7759/cureus.36653

**Published:** 2023-03-24

**Authors:** Radhika Thakur, Vakulabharanam Naga Rohith, Jainendra K Arora

**Affiliations:** 1 Surgery, Vardhman Mahavir Medical College and Safdarjung Hospital, Delhi, IND; 2 General Surgery, Vardhman Mahavir Medical College and Safdarjung Hospital, New Delhi , IND; 3 General Surgery, Vardhman Mahavir Medical College and Safdarjung Hospital, New Delhi, IND

**Keywords:** surgical mortality, sofa score, apache-ii score, sepsis, mortality rate in sepsis

## Abstract

Background: Sepsis is a medical and surgical emergency that describes the body’s systemic immunological response to an infectious process that can lead to end-stage organ dysfunction and death. Various clinical and biochemical parameters serve as indicators of organ dysfunction in patients with sepsis. Most familiar among them are the Sequential Organ Failure Assessment (SOFA) score, Acute Physiology and Chronic Health Evaluation (APACHE) II score, Mortality Prediction Score (MPM), and Simplified Acute Physiology Score (SAPS).

Methodology: A comparative study of APACHE II and SOFA scores was done at the time of admission in a total of 72 patients with sepsis and compared with the mean SOFA score. In our study, the SOFA score was measured serially and the mean SOFA score was calculated. All patients were selected according to the definition of sepsis (Sepsis-3). The ROC curve, the sensitivity, and the specificity were calculated to analyze the diagnostic value of SOFA, APACHE II, and the mean SOFA score. For all statistical tests, a “p-value” less than 0.05 was taken to indicate a significant difference.

Results: Our study showed that the mean SOFA score had a sensitivity of 93.65 and a specificity of 100, and on comparing the AUC of mean SOFA with APACHE II (Day 1) and SOFA (Day 1) - we got the P-value 0.0066 and 0.0008, which shows a statistically significant difference. So, we can say that the mean SOFA score is better than D_1 _(day 1 of admission) APACHE II & SOFA scores in predicting mortality in surgical patients with sepsis.

Conclusions: APACHE II and SOFA scores are equally effective in assessing mortality in surgical patients with sepsis at the time of admission. However, if we take serial measurements of SOFA scores and calculate the mean SOFA score it becomes a very useful tool for predicting mortality.

## Introduction

“Sepsis-3.0” defines sepsis as a life-threatening organ dysfunction caused by the dysregulated host response to infection [1,2] and “septic shock” in a patient who has serum lactate level of ≥2 mmol/L and requires vasopressor to maintain a MAP of ≥65mmHg, resulting in a higher risk of mortality [3-5]. Various studies have shown APACHE II [6] and SOFA [7] scores were independent prognostic factors for patients with sepsis. For the past 30 years, various scoring systems were developed for critically ill patients. Most familiar among them are the Sequential Organ Failure Assessment (SOFA) score, the Acute Physiology and Chronic Health Evaluation score (APACHE II) [6], the Mortality Prediction Model (MPM) [8], and the Simplified Acute Physiology Score (SAPS) [9]. They allow quantification of the severity of illness and the probability of in-hospital mortality. This allows for identifying the weakness and initiating interventions aimed at quality improvement.

Ganar et al. [10] conducted a prospective observational study in 2019 on 50 patients to assess and analyze the effectiveness of two prognostic scoring systems in predicting the mortality of critically ill patients in adult intensive care units. They found out that the SOFA score is better than APACHE 2 score to predict mortality in ICU patients.

To predict survival in patients with septic shock, FadiAlsous, Khamiees et al. [11] applied APACHE II and SOFA scores on 36 patients admitted to ICU with septic shock. Patients ranged in age from 16 to 85 years with a mean age of 67.4 ± 3.3 years. The mean admission APACHE II score was 25.4 ± 1.4, and the day 1 SOFA score was 9.0 ± 0.8. The nonsurvivors had higher mean APACHE II scores (29.8) than survivors (20.4) and higher first-day SOFA scores than survivors (10.8 v/s 6.9, respectively). The objective of our study was to predict mortality in surgical patients with sepsis using APACHE II and mean SOFA scores and comparison between the two scores.

## Materials and methods

This study was conducted at the Department of General Surgery, Vardhman Mahavir Medical College and Safdarjung Hospital, New Delhi from October 2018 to March 2020 after obtaining approval from the Institutional Ethics Committee (IEC) and the Review Board Committee (approval number: IEC/VMMC/SJH/Thesis/October/2018-191).

In this study, we aimed to predict mortality in surgical patients with sepsis using the APACHE II score at the time of admission and compare it with the mean SOFA score.

Inclusion criteria included patients above 12 years of age and patients with sepsis and exclusion criteria included patients less than 12 years of age.

A comparative study of APACHE II and SOFA scores was done at the time of admission in a total of 72 patients with sepsis and compared with the mean SOFA score. APACHE II is the sum of three units: an Acute Physiology Score, a chronic health evaluation, and a score based on the patient’s age [6]. It includes 12 physiological variables. APACHE II score is applied within 24 hours of hospital admission. APACHE II score varies from zero “0” to 71 points: up to 60 for physiological variables, up to 6 for age, and up to 5 for previous health status. In the SOFA score [7], we measure six organ systems depending on the level of dysfunction: respiratory, circulatory, renal, hematological, hepatic, and central nervous systems are taken into account, and the function of each is scored from 0 (normal functions) to 4 (most abundant).

In our study, the SOFA score was measured serially and the mean SOFA score was calculated. All patients were selected according to the new definition of sepsis (Sepsis-3). The ROC curve, the sensitivity, and the specificity were calculated to analyze the diagnostic value of SOFA, APACHE II, and the mean SOFA score. For all statistical tests, a “p-value” less than 0.05 was taken to indicate a significant difference. 

Statistical testing was conducted with the statistical package for the social science system version SPSS 25.0. Continuous variables were presented as mean±SD or median (IQR) for non-normally distributed data. Categorical variables were expressed as frequencies and percentages. The comparison of normally distributed continuous variables between the groups was performed using Student’s t-test. Nominal categorical data between the groups (survived and died) were compared using the Chi-squared test or Fisher’s exact test as appropriate. Non-normal distribution continuous variables were compared using the Mann-Whitney U test. A receiver operating characteristics (ROC) analysis was calculated to determine the optimal cut-off value of SOFA, APACHE II for predicting mortality. The area under the curve, the sensitivity, and the specificity was also calculated to analyze the diagnostic value of SOFA, APACHE II. For all statistical tests, a p-value less than 0.05 was taken to indicate a significant difference.

## Results

The study was done on a total of 72 surgical patients with sepsis. The majority of patients belonged to the age group of 21 to 30 with an age distribution of 27.78% followed by the age group of 51-60 years with an age distribution of 26.39%. Out of 72 patients, 23 were female and 49 were male. There was male preponderance in our study group with a male distribution of 68.06%.

Among our study group, 30 cases were of acute abdomen, 19 of necrotizing soft tissue infection, three gas gangrene, two meleneys' gangrene, two wet gangrene, three cholangitis, three Fournier’s gangrene, one ruptured hydatid cyst, two road traffic accidents, one gangrenous ileostomy, two cellulitis lower limb, and three ruptured liver abscess. All patients were selected according to the new definition of sepsis (Sepsis-3).

In our study group, 23 patients had comorbidities - diabetes mellitus (DM), hypertension (HTN), chronic kidney disease (CKD), chronic obstructive pulmonary disease (COPD), chronic liver disease (CLD), hepatitis B, hepatitis C, HIV, hepatocellular carcinoma (HCC), coronary heart disease, and hemolytic uremic syndrome (HUS). Most of the patients were on ionotropic support.

Out of 72 patients, there was a mortality of 63 patients (87.50%). Mean survival time of 3.68 ± 2.88 SD days, a median of three days, and IQR (Inter-quartile range) between one and six days. The survival time in days ranged between one and 10 days.

Out of the total study group of 72 patients, 63 patients had mortality and the mean APACHE II score for the patient who died is 20.76 ± 9.4SD and the median score is 21. The mean value for the APACHE II score on day 1 is 0.004 which is significant (Table 1).

**Table 1:** Association of APACHE II score at day 1 with mortality.

APACHE II score at day 1	Alive (n=9)	Died (n=63)	Total	P value
Mean ± S.D	11.67 ± 5.36	20.76 ± 9.4	19.62 ± 9.46	0.004
Median (IQR)	11 (8-14)	21 (13-28)	17 (12.75-27)	
Range	6-20	5-43	5-43	

The mean SOFA score of the patients who had mortality is 9.56 ± 4.51SD and median SOFA score of 10. The Mann-Whitney U test was performed, and P-value came out to be 0.017, which was statistically significant (Table 2).

**Table 2:** Association of SOFA score at day 1 with mortality.

SOFA score at day 1	Alive (n=9)	Died (n=63)	Total	P value
Mean ± S.D	5.56 ± 3.5	9.56 ± 4.51	9.06 ± 4.58	0.017
Median(IQR)	5 (2-6)	10 (5-13)	10 (5-13)	
Range	2-11	2-17	2-17	

We also studied the mean SOFA score of the patient by observing the trend of the SOFA score in our study groups by following the patient for a period of 10 days. Our study showed that the mean SOFA score for the patient who died was 11.11 ± 3.51 SD and the median of the patient who was declared dead was 12 with IQR of 9.083-14. The P-value of the patient for the mean SOFA score is <0.0001 which was statistically significant (Table 3).

**Table 3:** Association of mean SOFA score with mortality.

Mean SOFA score	Alive (n=9)	Died (n=63)	Total	P value
Mean ± S.D	2.57 ± 1.33	11.11 ± 3.51	10.04 ± 4.36	< .0001
Median(IQR)	2.33 (1.5-3)	12 (9.083-14)	10.85 (7.075-13.458)	
Range	1.17-4.67	1-17	1-17	

AUC stands for “Area under the ROC Curve.” The value of AUC ranges from 0 to 1. A model whose predictions are 100% wrong has an AUC of 0.0; one whose predictions are 100% correct has an AUC of 1.0. In our study, the day 1 APACHE II score for assessing mortality in patients with sepsis has AUC of 0.798, standard error of 0.0692, 95% confidence interval of 0.687 to 0.884, the P-value <0.0001 which was statistically significant and cut-off more than 20. The SOFA score on day 1 has ROC curve 0.745, standard error 0.0735, 95% confidence interval 0.629 to 0.841, and cut-off >6 and p-value 0.0008, which was statistically significant. The mean SOFA score has area under the ROC curve (AUC) 0.97, standard error of 0.019, 95% confidence interval of 0.900 to 0.996, cut-off >4.6667 and P-value <0.0001, which was statistically significant (Table 4). 

**Table 4:** Receiver operating characteristic curve of APACHE II score and SOFA score for assessing mortality.

	Area under the ROC curve (AUC)	Standard Error	95% Confidence interval	P value	Cut off
Day 1 APACHE II score	0.798	0.0692	0.687 to 0.884	<0.0001	>20
SOFA score (Day 1)	0.745	0.0735	0.629 to 0.841	0.0008	>6
Mean SOFA score	0.97	0.019	0.900 to 0.996	<0.0001	>4.6667

The day 1 APACHE II score with sensitivity of 50.79 and specificity of 100 had positive predictive value (PPV) of 100 and negative predictive value (NPV) of 22.5. Day 1 SOFA score had sensitivity of 68.25, specificity of 77.78, positive predictive value of 95.6 and negative predictive value of 25.9. The mean SOFA score had sensitivity of 93.65, specificity of 100, and had highest negative predictive value of 69.2; however positive predictive value is 100 which is similar to that of day 1 APACHE II score (Table 5).

**Table 5:** Diagnostic test to find out sensitivity, specificity, PPV and NPV of APACHE II score and SOFA score for assessing mortality.

Diagnostic test	Sensitivity(95% CI)	Specificity(95% CI)	PPV(95% CI)	NPV(95% CI)
Day 1 APACHE II score	50.79(37.9 - 63.6)	100(66.4 - 100.0)	100(89.1 - 100.0)	22.5(10.8 - 38.5)
SOFA score (Day 1)	68.25(55.3 - 79.4)	77.78(40.0 - 97.2)	95.6(84.9 - 99.5)	25.9(11.1 - 46.3)
Mean SOFA score	93.65(84.5 - 98.2)	100(66.4 - 100.0)	100(93.9 - 100.0)	69.2(38.6 - 90.9)

On comparison of AUC curve of APACHE II and SOFA score on day 1 of admission for assessment of mortality showed p-value of 0.3312, which shows that both APCHE II and SOFA score have similar discriminative powers when evaluated at the time of admission (day 1) for predicting hospital mortality in surgical patients with sepsis. However, when we compare mean SOFA score with day 1 SOFA score and day 1 APACHE II score the value came out to be 0.0008 and 0.0066, which was statistically significant (Table 6).

**Table 6:** Comparison of area under the curve (AUC) of APACHE II and SOFA scores at day 1 and mean SOFA score.

Comparison of AUC	Difference between areas	Standard Error	95% Confidence Interval	P value
SOFA score v/s APACHE II score day 1	0.0529	0.0545	-0.0538 to 0.160	0.3312
SOFA score v/s mean SOFA score	0.225	0.0668	0.0940 to 0.356	0.0008
Mean SOFA score v/s APACHE II score day 1	0.172	0.0633	0.0478 to 0.296	0.0066

## Discussion

Sepsis is a systemic inflammatory response syndrome that occurs during severe infection. It remains a principal cause of death in critically ill patients [12]. Several critical care and infectious disease specialist and researchers have focused their effort on sepsis in an attempt to gain a better understanding of the pathophysiological basis of sepsis or to develop new methods for the diagnosis and treatment of sepsis. In our study, we tried to assess the mortality of surgical patients with sepsis, for which we used the predictive scoring system - APACHE II and SOFA scoring system.

The study group was selected according to the new definition of sepsis (Sepsis 3.0). We calculated the APACHE II and SOFA scores for the patients on the first day of admission. We calculated scores using the worst value of each parameter in a period of 24 hours.

Sepsis can affect any age group and in the present study of 72 patients the age group ranged between 14 and 88 years with most of the patients falling under the age group of 21 to 30 years with a mean age of 41.28 years. There was male preponderance in our study with a male distribution of 68.06% and the female distribution of 31.94%. Other similar studies conducted by Todi et al. [13] and Abhinandan et al. [14] have shown male preponderance with most of the patients in the fourth to fifth decade.

In the present study out of the total 72 patients, there was a mortality of 87.50% of the patients, which was quite high compared to other studies by Ganar et al. [10] and Grissom et al. [15]. The mean survival time in days was 3.86 ± 2.88 days. The association of the APACHE II score on day 1 with an assessment of mortality had a mean of 20.76 ± 9.4 for nonsurvivors and survivors 11.67 ± 5.36 with a P-value of 0.004 which was statistically significant. The association of SOFA score on day 1 had a mean SOFA of 9.56 ± 4.5 1 for nonsurvivors and for survivors 5.56 ± 3.5 with a P-value of 0.017, which was statistically significant. The AUROC curve for APACHE II and SOFA score on day 1 of admission was 0.798 and 0.745 with 95% confidence interval of (0.687-0.884) and (0.629 to 0.841) and P-value <0.0001 and 0.0008, which were statistically significant. This reflects that the scores APACHE II and SOFA scores have a similar potential to assess the mortality in surgical patients with sepsis. A comparative study conducted by Ratanarat et al. [16] also revealed a similar outcome.

 So our present study suggests that both SOFA score and APACHE II score have similar discriminative powers when evaluated at the time of admission (Day 1) for predicting hospital mortality in surgical patients with sepsis as there is no statistically significant difference between the two scores on day 1 with a P-value of 0.3312. However, the sensitivity of the APACHE II score in predicting mortality on day 1 is (50.79) which is less than that of the SOFA score on day 1 (68.25) but the specificity is higher (100) than the SOFA score on day 1 (77.78).

In our present study, we also took the serial measurement of the SOFA score in the study group for a period of 10 days. There was a statistically significant difference between the AUC of SOFA and the mean SOFA score (mean of serially measured SOFA scores) with a P-value of 0.0008, and the AUC of Day 1 APACHE II and mean SOFA score has a P-value of 0.0066 which was also statistically significant and the mean SOFA score had sensitivity 93.65 and specificity 100, depicting that mean SOFA is a better predictor of mortality than D1 APACHE II and D1 SOFA scores. Hence, the SOFA scoring system has higher predictive value when measured serially as the mean and highest scores reflect the patient’s clinical status more accurately. A previous study conducted by Ganar et al. [10] in 2019 also had similar results revealing SOFA score is a better predictor of mortality than the APACHE 2 score in critically ill patients.

So, from our study, we have been able to reveal that SOFA scoring is as efficient as the APACHE II scoring system at the time of admission in the assessment of mortality in surgical patients with sepsis. However, if serial measurements of the SOFA score are done during the patient’s hospital admission, and the mean SOFA score is taken, it becomes a very useful tool in predicting mortality.

## Conclusions

Sepsis is a major healthcare problem and one of the leading causes of mortality and morbidity among surgical patients. The results from our study show that both APACHE II and SOFA scores are equally effective in assessing mortality in surgical patients with sepsis at the time of admission; however, if serial evaluation of the SOFA score is done and the mean of SOFA score calculated to assess outcome it gives better results, with the trend of SOFA score declining in survivors while nonsurvivors had a stable higher score.

## Appendices

**Table 7:** APACHE II score. Chronic Health Problems: Cirrhosis of the liver (confirmed by biopsy) New York Heart Association Class IV Severe COPD- Hypercapnia,home O2 use, or pulmonary hypertension On regular dialysis Immunocompromised None (0 points) Non- Surgical (5 points) Emergent operation (5 points) Elective operation (2 points)

Variables	4	3	2	1	0	1	2	3	4
Temperature (^0^C)	≥41	39-40.9		38.5-38.9	36-38.4	34-35.9	32-33.9	30-31.9	≤29.9
MAP- mmHg	≥160	130-159	110-129		70-109		50-69		≤49
Heart Rate	≥180	140-179	110-139		70-109		55-69	40-54	≤39
Respiratory rate	≥50	35-49		25-34	12-24	10-11	6-9		≤5
A-aPO2 (mmHg) FiO2>50%OR PaO2(mmHg) (FiO2<50%)	≥500	350-499	200-349		<200 PO_2_>70	PO_2_ 61-70		PO_2_ 55-60	PO_2_ <55
pH or HCO3 (arterial pH is preferred)	Ph≥7.7 HCO3 ≥52	7.6-7.69 41-51.9		7.5-7.59 32-40.9	7.33-7.49 22-31.9		7.25-7.32 18-21.9	7.15-7.24 15-17.9	<7.15 <15
Serum sodium (mEq/l)	≥180	160-179	155-159	150-154	130-149		120-129	111-119	≤110
Serum potassium (mEq/l)	≥7	6-6.9		5.5-5.9	3.5-5.4	3-3.4	2.5-2.9		<2.5
Serum Creatinine (mg/dl) Double point score for acute renal failure	≥3.5	2-3.4	1.5-1.9		0.6-1.4		<0.6		
Hematocrit (%)	≥60		50-59.9	46-49.9	30-45.9		20-29.9		<20
WBC count (total/mm^3^)	≥40		20-39.9	15-19.9	3-14.9		1-2.9		<1
Glasgow coma score Score=15 minus actual GCS	11	12	13	14	15				
	6	5	3	2	0				
Age(years)	<44=0; 45 to 54=2; 55 to 64=3; 65 to 74=5; >75=6								

**Table 8:** SOFA score.

Variables	0	1	2	3	4
Respiratory PaO2/FiO2 mmHg	>400	≤400	≤300	≤200 with respiratory support	≤100 with respiratory support
Renal Creatinine, mg/dL or Urine output	<1.2	1.2-1.9	2.0-3.4	3.5-4.9 <500	≥5.0 <200
Serum bilirubin mg/dL	<1.2	1.2-1.9	2.0-5.9	6.0-11.9	≥12.0
Cardiovascular Hypotension	NO	MAP <70mmHg	Dop ≤5 or Dob (any dose)	DA >5 or Epi ≤0.1 or NE ≤0.1	DA >15 or Epi>0.1 or NE >0.1
Coagulation Platelets×10^3^/µL	>150	≤ 150	≤100	≤50	≤20
GCS	15	13-14	10-12	6-9	<6
Dob-dobutamine; Dop-dopamine; Epi-epinephrine; Norepi-norepinephrine. Adrenergic agents were infused for at least 1 hour (doses given are in µg/kg per minute)					

## References

[1] Singer M, Deutschman CS, Seymour CW (2016). The third international consensus definition for sepsis and septic shock (Sepsis-3). JAMA.

[2] Seymour CW, Liu VX, Iwashyna TJ (2016). Assessment of clinical criteria for sepsis: for the third international consensus definition for sepsis and septic shock (Sepsis-3). JAMA.

[3] Shankar-Hari M, Phillips GS, Levy ML (2016). Developing a new definition and assessing new clinical criteria for septic shock: for the third international consensus definitions for sepsis and septic shock (Sepsis-3). JAMA.

[4] Vincent JL, de Mendonça A, Cantraine F (1998). Use of the SOFA score to assess the incidence of organ dysfunction/failure in intensive care units: results of a multicenter, prospective study. Working group on "sepsis-related problems" of the European Society of Intensive Care Medicine. Crit Care Med.

[5] Kaukonen KM, Bailey M, Pilcher D, Cooper DJ, Bellomo R (2015). Systemic inflammatory response syndrome criteria in defining severe sepsis. N Engl J Med.

[6] Knaus WA, Zimmerman JE, Wagner DP, Draper EA, Lawrence DE (1981). APACHE-acute physiology and chronic health evaluation: a physiologically based classification system. Crit Care Med.

[7] Vincent JL, Moreno R, Takala J (1996). The SOFA (Sepsis related organ failure assessment) score to describe organ dysfunction or failure on behalf of the working group on sepsis related problems of the European Society of Intensive Care Medicine. Intensive Care Med.

[8] Lemeshow S, Teres D, Avrunin JS, Gage RW (1988). Refining intensive care unit outcome prediction by using changing probabilities of mortality. Crit Care Med.

[9] Le Gall JR, Lemeshow S, Saulnier F (1993). A new simplified acute physiology score (SAPS) based on a European/North American multicenter study. JAMA.

[10] Ganar A, Potey S (2019). To evaluate and compare ability of two prognostic scoring systems in predicting the mortality of critically ill patients in adult intensive care unit. IJCMR.

[11] Alsous F, Khamiees M, DeGirolamo A, Amoateng-Adjepong Y, Manthous CA (2000). Negative fluid balance predicts survival in patients with septic shock: a retrospective pilot study. Chest.

[12] Hotchkiss RS, Karl IE (2003). The pathophysiology and treatment of sepsis. N Engl J Med.

[13] Todi S, Chatterjee S, Sahu S, Bhattacharyya M (2010). Epidemiology of severe sepsis in India: an update. Crit Care.

[14] Abhinandan KS, Vedavathi R (2013). “Usefulness of sequential organ failure assessment (SOFA) and acute physiology and chronic health evaluation II (APACHE II) score in analyzing patients with multiple organ dysfunction syndrome in sesis”.. J Evolut Med Dental Sci.

[15] Grissom CK, Brown SM, Kuttler KG, Boltax JP, Jones J, Jephson AR, Orme JF Jr (2010). A modified sequential organ failure assessment score for critical care triage. Disaster Med Public Health Prep.

[16] Ratanarat R, Thanakittiwirun M, Vilaichone W, Thongyoo S, Permpikul C (2005). Prediction of mortality by using the standard scoring systems in a medical intensive care unit in Thailand. J Med Assoc Thai.

